# Impact of New Orleans Saints Football Games on Internal Medicine Admissions

**DOI:** 10.31486/toj.20.0137

**Published:** 2021

**Authors:** Vu D. Nguyen, Chayan Chakraborti

**Affiliations:** Department of Medicine, Tulane University School of Medicine, New Orleans, LA

**Keywords:** *Football*, *internal medicine*, *New Orleans*, *patient admission*, *treatment adherence and compliance*

## Abstract

**Background:** Various studies have reported a decrease in emergency department admissions in correlation with major sporting events. However, these studies limit the investigation of selected variables to the 24-hour periods on game days. For our study, we assessed the impact of New Orleans Saints football team games on medical admissions on game days and also on post-game days.

**Methods:** We collected Saints game results from the 2013-2014 to 2019-2020 season and admissions data from 3 local hospitals during the same 7-year period. We compared the average daily census of game days vs non-game days as our control group using a 2-sample *t* test with unequal variances.

**Results:** The results demonstrate a statistically significant reduction in the average number of internal medicine admissions on game days (19.6%, *P*=0.0001). This reduction in admissions was not observed on post-game days (5.84%, *P*>0.05). On post-game days following a Saints victory, we found a statistically significant increase in the average number of admissions compared to when the team lost the previous day (18.2%, *P*=0.0105).

**Conclusion:** To our knowledge, the relationship between a recurring major sporting event and inpatient internal medicine admissions has not been previously quantified. Our study of the impact of the New Orleans Saints football games on internal medicine admissions provides insights into the relationship between social sporting events and health outcomes in the city. While our results may not be directly generalizable to other locations—particularly those that are not in a National Football League team market—we showed that during a 7-year period, Saints football games correlated with a 19.6% decrease in internal medicine admissions on the day of the game. This finding suggests that Saints games may serve as a protective factor associated with decreased negative health outcomes among residents in the city.

## INTRODUCTION

Despite remarkable biomedical advancements and great efforts in clinical care, clinical care is estimated to account for only 20% of a community's overall health outcomes. The remaining factors are directly related to health behaviors (30%), social and economic factors (40%), and physical environment (10%).^1^ Observing patterns of health outcomes with respect to patients’ behaviors and interactions with their surroundings—such as at a sporting event—can lead to a greater appreciation of the impact of social events on a community's health.

Studies support a correlational relationship between sporting events and the perceived health outcomes of various patient populations across different geographic regions of the world. In Japan, Inoue et al reported a positive association between sport spectatorship and the perception of general health through their analysis of 12-year cross-sectional data collected from national surveys.^2^ Specifically, in comparison to those who did not attend any sporting event in a year, those who did were 33% more likely to report a higher level of self-rated health. The study suggests that attending sporting events is an important nonexercised form of leisure that might produce psychosocial resources and contribute to good health. Similarly, Almeida et al demonstrated a reduction in the number of patients coming to the emergency department (ED) in Portugal during major sport events such as soccer games, especially patients with less severe presentations.^3^

Compared to countries such as Japan and Portugal, the patient populations in the United States are larger and more diverse in terms of social ethnicities and clinical pathologies. Depending on geographic locations and socioeconomic factors, patients in the United States have different levels of access to health care, hold unique sociocultural beliefs, and exhibit distinctive behaviors in their physical environments and social support groups. Cultural differences also exist with respect to sports. While American football is only the ninth most popular sport in the world, it is the most popular sport in the United States and serves not only as a source of entertainment but also as a bonding agent between many people and their communities.^4^ An average of 16.5 million Americans watched National Football League (NFL) games during the 2019 season, and the New Orleans Saints had an average of 73,082 fans at each home game in 2019, making the Mercedes-Benz Superdome, home stadium of the New Orleans Saints, the seventh most popular NFL venue.^5,6^

Reich et al found a significant decrease in ED admissions during broadcasts of American football Super Bowl finals from 1988 to 1992.^7^ The authors suggested that the decline in ED admission rates may be even more prominent when the local team is one of the teams participating in the event. However, the study looked only at a single Super Bowl championship game in each season, which is played at a neutral stadium and always receives tremendous interest from many football fans outside of the 2 participating teams’ supporter bases.

As of December 2020, we had not found a study assessing the relationship between a local professional sports team and the number of inpatient admissions on the game day or the day after the game. In this retrospective study, we investigated the impact of New Orleans Saints football team games on internal medicine admissions at 3 local hospitals during game days and post-game days during a 7-year time period from 2013-2020.

## METHODS

The institutional review board determined this study to be exempt. We obtained historical data about the New Orleans Saints football team from publicly available databases that report game results.^8^ This data included the team record from the 2013-2014 season through the 2019-2020 season. Deidentified admissions data from 3 Tulane University School of Medicine–affiliated hospital systems in New Orleans during the same 7-year period (2013-2020) were reported as aggregate daily admissions to the Tulane Internal Medicine Service. No individual patient data were included in this study; only the aggregate admission census numbers were analyzed. All 3 hospitals are located <2 miles from the Mercedes-Benz Superdome. The day of admission was defined by the time the admission order was recorded in the hospital electronic medical record. Admissions within a 24-hour period from 7:00 am to 6:59 am the next day were counted as same day.

We analyzed the relationship between the record of the New Orleans Saints and internal medicine admissions. Variables included game outcome (win/loss), game location (home/away), game type (regular season/playoff), and day type (game day/post-game day/no game). Additional categories created were in-conference (National Football Conference [NFC] South) games vs games involving nonconference teams and rivalry games, defined as games vs the Atlanta Falcons.

We compared the average daily census of game days vs non-game days (excluding post-game days) using a 2-sample *t* test with unequal variances. All statistical analyses were performed using Stata, release 11.2 (StataCorp LP).

## RESULTS

We obtained complete data for 87.0% of non-game days (1,269 of 1,458), 85.6% of game days (89 of 104), and 88.5% of post-game days (92 of 104). The Table shows the average number of admissions by New Orleans Saints football game variables. In general, on game day, we found a statistically significant reduction (19.6%) in the average number of internal medicine admissions to the 3 local hospitals (23.39 vs 29.10, *P*=0.0001). However, on post-game days, average admissions increased (5.84%; 30.8 vs 29.10, *P*>0.05).

**Table.** Mean Number of Internal Medicine Hospital Admissions by New Orleans Saints Football Game Variables

Time Period	Variable	Days, n	Average Admissions	SD	*P* Value
Overall 2013-2020	No game	1,269	29.10	10.04	
	Game day	89	23.39	9.0	0.0001
	Post-game day	92	30.8	11.23	>0.05
Game days only	Win	55	22.91	8.55	0.522
	Loss	34	24.18	9.78	
	vs NFC South opponents	28	22.21	7.41	0.405
	vs non–NFC South opponents	61	23.93	9.66	
	vs Falcons	9	22.67	5.66	0.80
	vs other non-Falcons opponents	80	23.48	9.33	
Post-game days only	Win	55	33.24	11.06	0.0105
	Loss	37	27.19	10.61	
	vs NFC South opponents	28	31.39	12.65	0.741
	vs non–NFC South opponents	64	30.55	10.64	
	vs Falcons	8	32.13	9.26	0.73
	vs other non-Falcons opponents	84	30.68	11.43	

NFC, National Football Conference.

On game days, the variables win/loss, playing against NFC South/non–NFC South opponents, and playing against Falcons/non-Falcons opponents had no effect on the average medical admissions rate. On the other hand, on post-game days following a Saints victory, we found a statistically significant increase (18.2%) in the average number of admissions compared to when the Saints lost the previous day (33.24 vs 27.19, *P*=0.0105). The other variables—playing against NFC South/non–NFC South opponents and against Falcons/non-Falcons opponents—did not demonstrate any correlational relationship with average hospital admissions on post-game days.

We found no statistically significant differences in the number of average hospital admissions on game days or post-game days for the additional variables studied but not included in the Table: home vs away games and preseason vs regular season vs playoff games.

## DISCUSSION

The concept of social determinants of health is crucial in establishing a holistic understanding of the interrelationship among individuals, communities, and health outcomes. Factors such as socioeconomic status, social environments, and barriers to care should be taken into consideration in assessing the health profiles and psychosocial behaviors of a patient population. The city of New Orleans and its people provide an example of the complexity of clinical care delivery to underserved patient populations in a multisociocultural environment. As one of the oldest cities in America, New Orleans has a fascinating history, rich culture, and unique social activities that bring its communities together, one of which is watching Saints games.

The Dixit et al study (2019) concluded that weather conditions in New Orleans had no significant associations with medical admission rates.^9^ While the study primarily looked at environmental triggers, our retrospective study focused on the impact of a recurring sporting event on the health care system in our city. From our results, we can reasonably conclude that total admissions to internal medicine wards decrease by 19.6% on game days. This finding is consistent with our hypothesis and other studies discussed previously.^2,3,7^

An additional finding from our study is that Saints football games do not exclusively affect average hospital admissions on game days, but this impact extends to the 24 hours after the game as well. Specifically, on the post-game day following a Saints victory, we found an 18.2% increase in the average number of admissions compared to the average census following a Saints loss. This observation suggests that the Saints winning their games may correlate with a delay in seeking care and predispose more patients requiring admission on the following day. Taking the local sociocultural context and health profiles of our patient populations into consideration, these phenomena can be potentially explained.

A major part of the New Orleans culture is our affinity to engage in social activities that involve community participation, such as Mardi Gras, the New Orleans Jazz & Heritage Festival, and many other festivals and events. The mental health and overall wellness of our population are certainly benefited by this nurturing system of community unity and social support. Similarly, attending the Saints games or watching the team play on TV is a bonding experience for many people and families here, which may serve as a protective factor conductive to health outcomes.

Furthermore, New Orleans is a carnival city deeply rooted in the culture of celebration, encapsulated by the unofficial city motto “laissez les bons temps rouler,” meaning “let the good times roll.” For many, this mentality involves eating rich foods, smoking, drinking, and revelry—often to excess. According to the New Orleans Community Health Improvement Report, among the 343,829 residents in Orleans Parish, 21% of adults smoke, 19% excessively drink, and 30% are obese, compared to the national benchmarks of 14%, 8%, and 25%, respectively. Also of note is that 21% of residents here have limited access to healthy foods.^10,11^

In the setting of these lifestyle risk factors, the having-a-good-time mindset may be especially prevalent whenever the Saints play, manifested in large tailgate parties and big-crowd gatherings before, during, and after each game. When our beloved team wins, fans are not shy about celebrating, which often translates into lack of self-care, engaging in risky activities, and overlooking strict adherence to recommended medical treatment. We hypothesize that while watching the Saints games could be an innate protective factor, this positive influence alone might not be sufficient to maintain adequate clinical stability when patients indulge in excessive celebrations after each Saints win, likely leading to avoidable injuries and acute exacerbations of chronic diseases such as congestive heart failure, chronic kidney disease, and chronic obstructive pulmonary disease. However, further in-depth studies are warranted to provide support for this theory.

The most important limitation in our study is that we did not include the details specific to each admission or the principal diagnoses. A thorough analysis of these variables would give us more confidence in our hypothesis that the etiology of most admissions on game days and post-game days is related to acute decompensation of preexisting conditions.

Another notable study limitation is that our data were collected from only 3 hospitals located within 2 miles of the Mercedes-Benz Superdome in downtown New Orleans. The Superdome had an average of >73,000 fans in attendance in 2019, and game-related traffic could potentially play a role in deterring patients to seek care at these facilities.^6^ While traffic is a common occurrence around sporting events, heavy traffic congestion may also be a function of a temporary increase in the New Orleans population. We were not able to directly account for the contribution of visitors to the city with respect to the increased census numbers. However, we believe that including non-game days in our analysis served as an adequate control for the potential confounder.

Moreover, our design did not take into consideration the time of day for each football game. Each NFL game typically lasts 3.5 to 4 hours, and most games are played in the afternoon and early evening. Our cutoff time for admission day may not have corresponded well with when patients presented to the facilities. Our admission tally for a given day ends at 6:59 am the next day, so those who watch or attend games that take place later in the evening could possibly arrive at the ED relatively later and be counted as part of the post-game day census. To account for this trend, we chose to also examine the post-game day census.

Last, we focused on admissions to 3 hospitals proximate to the Mercedes-Benz Superdome rather than emergency room visits. While instances of excess indulgence (eg, raucous victory celebrations, drowning sorrows after a loss) were strong possibilities behind the observed change in admission rates, alternative explanations—including medication nonadherence, clinic closures on weekends, traffic patterns, and public transportation limitations—may have also contributed. However, interruptions in these services are likely because of the summative effect that a professional football game has on a locality, and as such, we believe that these factors may have been minimized by our examination of non-game days as the control.

## CONCLUSION

The relationship between a recurring major sporting event and inpatient internal medicine admissions has not been previously quantified. Our study of the impact of the New Orleans Saints football games on internal medicine admissions provides insights into the relationship between social sporting events and health outcomes in the city. While our results may not be directly generalizable to other locations—particularly those that are not in an NFL team market—we showed that during a 7-year period from 2013-2020, Saints football games correlated with a 19.6% decrease in internal medicine admissions on the day of the game. This finding suggests that Saints games may serve as a protective factor associated with a decreased negative health outcome among residents in the city.

The results of this study can help hospital administrators and city planners optimize staff schedules and allocate resources to respond to specific needs of patient populations. In addition, this study can potentially contribute to modifications of public health policies to address the nuances of social determinants of health in New Orleans, such as improving public transportation, increasing access to physical and behavioral health care, and encouraging healthy lifestyles.

Future studies are needed to determine the magnitude of the spectator sports effect on admissions in other cities and whether this effect is preserved in America across sporting events other than NFL games.

## Notes

*This article meets the Accreditation Council for Graduate Medical Education and the American Board of Medical Specialties Maintenance of Certification competencies for Patient Care and Medical Knowledge.*
